# Synthesis of Naringenin and Senecioic Acid Ester Derivatives and Biological Evaluation of the Astrocyte Antioxidant Mechanism and Reactivity After Inflammatory Stimulus

**DOI:** 10.3390/ijms26052215

**Published:** 2025-02-28

**Authors:** Janaína Ribeiro Pereira Soares, Cleonice Creusa dos Santos, Lucas Matheus Gonçalves de Oliveira, Heráclito Rocha Neto, Maurício Moraes Victor, Elivana Lima França, Maria de Fátima Dias Costa, Silvia Lima Costa, Juciele Valeria Ribeiro de Oliveira

**Affiliations:** 1 Laboratory of Neurochemistry and Cellular Biology, Institute of Health Sciences, Federal University of Bahia, Av. Reitor Miguel Calmon S/N, Salvador 40231-300, Brazil; janainaribeirolabnq@gmail.com (J.R.P.S.); cleonicemev@gmail.com (C.C.d.S.); lucas.nom@gmail.com (L.M.G.d.O.); heraclito.neto@ufba.br (H.R.N.); fatima@ufba.br (M.d.F.D.C.); 2 Department of Organic Chemistry, Institute of Chemistry, Federal University of Bahia, Salvador 40170-115, Brazil; mmvictor@ufba.br; 3 Federal Institute of Bahia, Campus Vitória da Conquista, Vitória da Conquista 45078-300, Brazil; elivana.franca@ifba.edu.br; 4 National Institute of Translational Neuroscience (INNT), Rio de Janeiro 21941-902, Brazil

**Keywords:** *Citrus paradisi*, naringenin, senecioic acid esters flavonoids, astrocytes, antioxidants

## Abstract

The imbalance between the overproduction of reactive species and antioxidant mechanisms can result in astrogliosis and oxidative stress associated with neurodegeneration. Based on the described antioxidant activity of naturally occurring flavonoids, this study evaluated the antioxidant mechanisms of the flavonoid naringenin and the senecioic acid ester derivatives in cortical astrocytes. Naringenin and (*S*)-naringenin were purified from *Citrus paradisi*, and from them 7,4-*O*-disenecioic ester naringenin, (*S*)-7,4-*O*-disenecioic ester naringenin, and 7-*O*-senecioic ester naringenin were synthesized and tested for antioxidant activity by the free-radical scavenging reaction with DPPH. The flavonoids’ toxicity and glutathione (GS) depletion were determined in rat astrocyte cultures; the effects on the astrocytes’ reactivity was determined by the expression of the glial fibrillary acidic protein (GFAP) and by measuring nitric oxide (NO) production in astrocytes treated with lipopolysaccharide (LPS, 1 µg/mL/24 h). The compounds (1–10 μM) presented antioxidant effects, and the (*S*)-7,4′-*O*-disenecioic ester naringenin was the most effective. The compounds (1–100 μM) were not toxic to the astrocytes, also promoting an antioxidant effect by increasing GSH. Moreover, naringenin, (*S*)-7,4′-*O*-disenecioic ester naringenin, and 7-*O*-senecioc ester naringenin mitigated the astrocyte reactivity induced by LPS, reducing GFAP expression and NO production. These findings indicate that naringenin and senecioic acid ester derivatives present a pharmacological potential as antioxidant and anti-inflammatory compounds for brain diseases via the modulation of astrocyte response.

## 1. Introduction

Astrocytes play a significant role in controlling homeostasis in the central nervous system and are the first line of defense against brain injury, energy imbalance, and oxidative stress [1]. A large body of research shows that astrocytes also play vital roles in the neuroinflammatory processes associated with aging and neurodegenerative diseases [1,2]. Astrocytes maintain a complex enzymatic antioxidant system capable of protecting neurons from oxidative stress involving superoxide dismutase (SOD), also supplying cysteinylglycine (CysGly) as a precursor for glutathione (GSH) and some phase II reaction enzymes [3]. However, under some dysfunctional conditions such as aging and neurodegenerative diseases, the astrocytes enter in a state of activation known as reactive astrogliosis, and the antioxidant system is overcome, so it is essential to supplement the diet with molecules that, in part, control reactivity and bolster the astrocytes’ antioxidant self-defenses and neuroprotective activities [4,5].

Flavonoids are a class of oxygen heterocyclic compounds that are products of the secondary metabolism of plants and are responsible for the aroma and color of flowers, fruits, and vegetables, playing essential protective roles in plants against different kinds of stresses. In the last decades, flavonoids have been considered for their therapeutic potential as neuroprotective agents [6,7]. Among these, the flavanone naringenin, abundant in citrus fruits, has been the subject of investigation about its antioxidant effects and neuroprotective potential. Naringenin showed dose-dependent protection against oxidative damage to liver lipids with a reduction in DNA damage but no protective effect on the oxidation of GSH [8]. In the study by Hassan et al. [9], the neuroprotective effect of naringin against cerebellar changes in an in vivo Alzheimer’s disease model was demonstrated through the modulation of autophagy, oxidative stress, and tau expression. As recently revised by Atoki et al. [10], naringenin has the ability to impact a number of signaling pathways, suggesting that it may be useful as a neurotherapeutic drug; however, one limitation of its use is its low lipophilicity, being poorly absorbed in the human gastrointestinal tract after oral dosing.

Acid esters increase flavonoids’ lipophilicity, favoring an increased affinity for biological membranes and better interactions with target proteins such as prenylated flavonoids, a subclass of flavonoids that combine a flavonoid skeleton with a lipophilic prenyl side chain [11,12]. A study by Mai et al. (2020) [13] highlighted the cytotoxic activity of the natural flavonoid 6,8-diprenyl-4′-methyl-naringenin, isolated from the fruits of *Macaranga balansae*, against cancer cell lines. To date, prenylation increases the lipophilicity of flavonoids, which results in a high biological activity, as demonstrated by Santos et al. (2020) [14] and, more recently, by Lv et al. (2023) [15], which revised the importance of prenyl groups for the antioxidant properties of flavonoids in several in vitro and in vivo models. However, naturally occurring compounds that combine the flavonoid portion and a side chain of the lipophilic groups are rare, which limits the application of these bioactive compounds in pharmaceuticals. Thus, synthesizing these molecules based on naturally occurring flavonoids is an excellent way to obtain these more lipophilic flavonoid derivatives, and many studies have been developed with the view to better purify naringenin and to generate more lipophilic derivatives [16,17,18]. Hence, in the present study, we investigated the toxicity, antioxidant properties, and mechanisms of the natural flavonoid naringenin and its esters’ derivatives in cultures of rat cortical astrocytes subjected to inflammatory damage.

## 2. Results

### 2.1. Naringenin and Senecioic Acid Ester Derivatives Characterized by Nuclear Magnetic Resonance (NMR)

**Figure 1:**
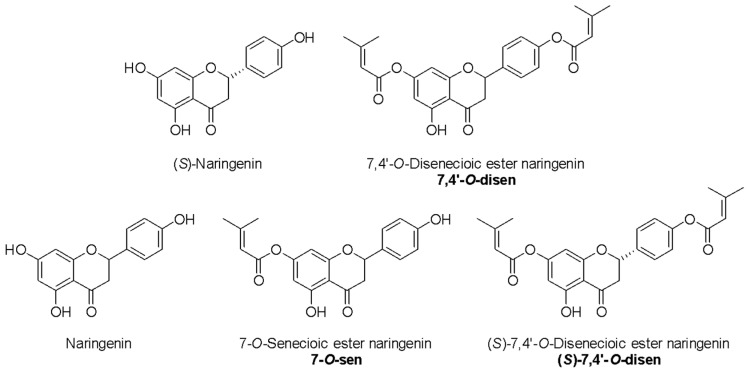
Chemical structure of flavanones naringenin, (*S*)-naringenin, 7,4′-*O*-disenecioic ester naringenin, (*S*)-7,4′-*O*-disenecioc ester naringenin, and 7-*O*-senecioc ester naringenin.

The flavonoid naringenin was extracted from the peel of *Citrus paradisi* (grapefruit) [18]. Through structural modifications of this flavonoid resulting from the esterification of the flavanone naringenin with senecioic acid, another three senecioic acid ester molecules were generated: 7-*O*-senecioc ester naringenin (7-*O*-sen); 7,4′-*O*-disenecioic ester naringenin (7,4′-*O*-disen), and (*S*)-7,4′-*O*-disenecioc ester naringenin ((*S*)-7,4′-*O*-disen) (Figure 1). The products were characterized by ^1^H and ^13^C nuclear magnetic resonance (NMR) (see the Supplementary Materials).

### 2.2. Antioxidant Effects of Naringenin and Senecioic Acid Ester Derivative Isomers

**Figure 2:**
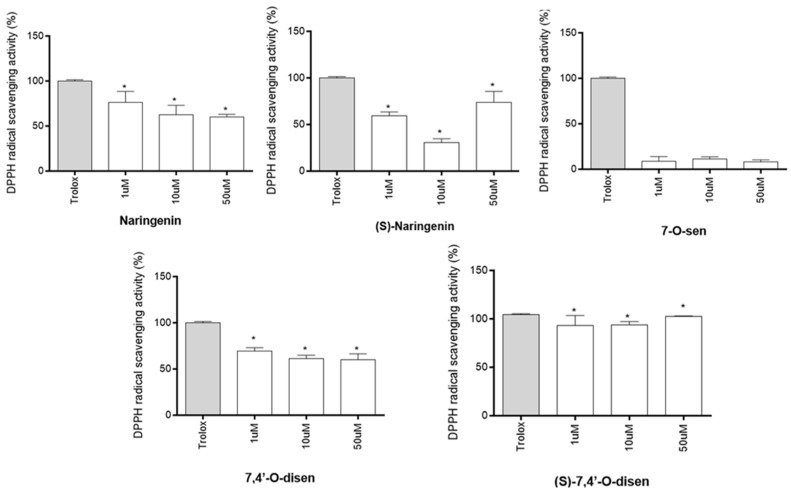
Antioxidant activity of flavonoids on DPPH radical scavenging over 15 min. Results are expressed as the mean ± SD of the percentage of DPPH radical scavenging activity in increasing concentrations of the flavonoids (at 1, 10, and 50 μM) compared with Trolox. Data were analyzed using One-way ANOVA followed by Tukey’s multiple comparison post-test. (*) indicates a statistical difference (*p* < 0.05) relative to the Trolox antioxidant activity, considered to be 100%.

We tested, first, if the flavonoids naringenin, its isomer (*S*)-naringenin, and the senecioic acid ester derivatives 7,4′-*O*-disen, (*S*)-7,4′-*O*-disen, and 7-*O*-sen have similar antioxidant capacities using the DPPH cell free assay at growing concentrations of 1, 10, and 50 µM. This test involves the elimination of the DPPH free radical and is one of the methods most used to screen compounds with antioxidant activity due to its low cost, speed, and accessibility for measuring the antioxidant properties of phenolic substances and extracts [19]. Among the flavonoids tested, four of them showed a capacity greater than 50% in eliminating free radicals at the concentration of 1 µM; these were (*S*)-7,4′-*O*-disen (93.10%), naringenin (76.06%), (*S*)-naringenin (59.19%), and 7,4′-*O*-disen (69.68%). On the other hand, 7-*O*-sen did not present antioxidant activity in this assay at any concentration tested. However, from the concentration of 10 µM, the antioxidant capacity of the compounds naringenin and 7,4′-*O*-disen were reduced but were still significant. Only (*S*)-7,4′-*O*-disen showed a capacity greater than 50% in eliminating free radicals in the three concentrations tested when compared with Trolox (standard) in the same concentrations (Figure 2).

### 2.3. Toxicity of Naringenin and Senecioic Acid Ester Derivatives to Astrocytes and GS Depletion

**Figure 3:**
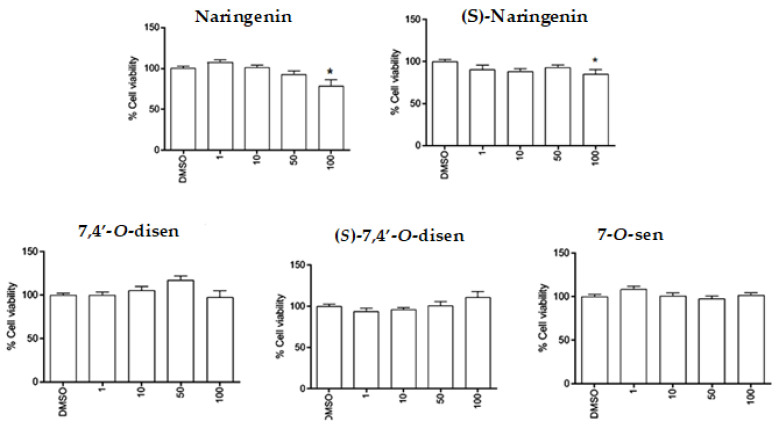
Evaluation of the toxicity of flavonoids and synthetic derivatives in primary culture of astrocytes through the MTT test. The activity of mitochondrial dehydrogenases was measured after 24 h after exposing the cells to molecules at concentrations of 1, 10, 50, and 100 µM or to 0.1% DMSO (vehicle). Data were analyzed using One-way ANOVA followed by Student–Newmann–Keuls post-test and Dunn’s post-test. Results expressed as mean ± SD as a percentage in relation to 0.1% DMSO considered as 100%. (*) indicates a statistical difference (*p* < 0.05) relative to control (0.1% DMSO).

With a view to characterizing the effects of the compounds on astrocyte viability, screening was carried out with the flavonoid naringenin, its isomer (*S*)-naringenin, and the senecioic acid ester derivatives 7,4′-*O*-disen, (*S*)-7,4′-*O*-disen, and 7-*O*-sen. This assay’s concentrations and treatment period were based on previous work with polyhydroxylated flavonoids [20,21]. The astrocytes were treated with flavonoids at 1, 10, 50, and 100 µM, and cell viability was assessed by the MTT test after 24 h treatments. Compared with the control cultures (DMSO 0.1%), the compounds were not toxic to the astrocytes at the concentrations tested, and only naringenin and (*S*)-naringenin presented a slight toxicity (less than 15%) to astrocytes at the highest concentration adopted (100 µM) (Figure 3). 

**Figure 4:**
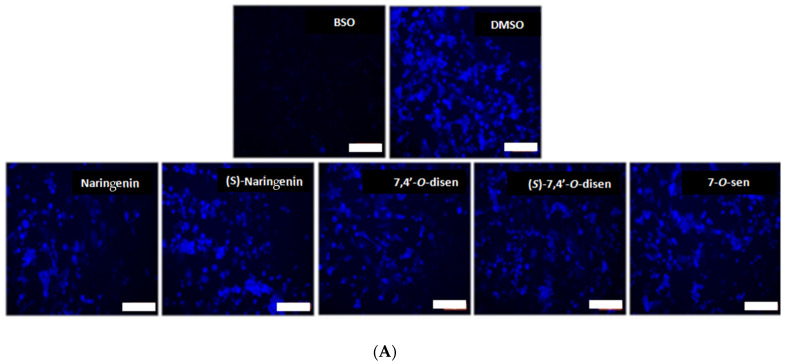

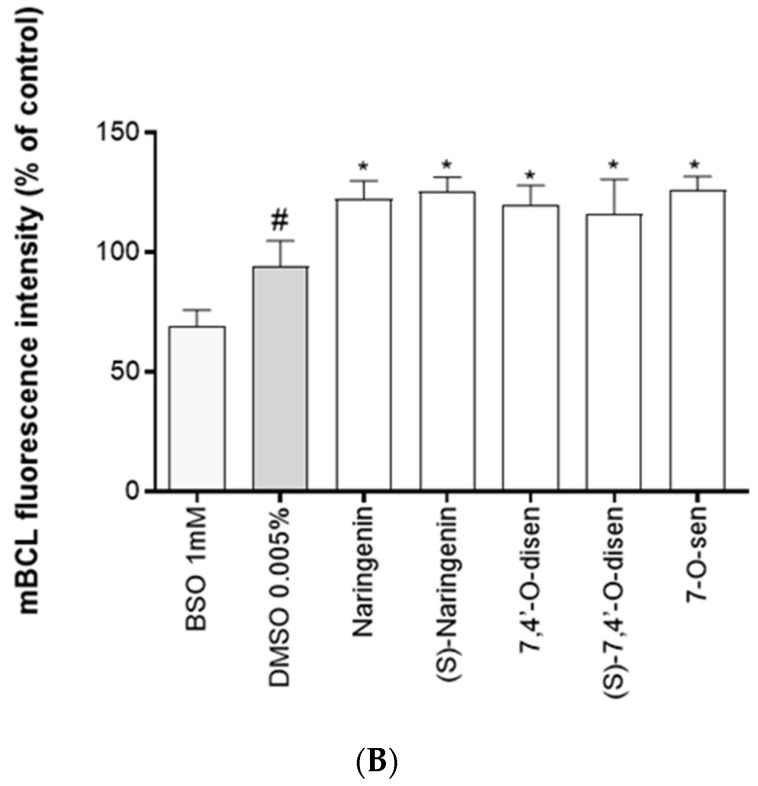
Effect of naringenin and senecioic acid ester derivatives on astrocytes’ glutathione (GSH) depletion using the monochlorobimane (MCB) test. (**A**) Images of astrocyte cultures 24 h after treatment with D,L-buthionine-(*S*,*R*)-sulfoximine (BSO, 1 mM) or with the flavonoids naringenin, (*S*)-naringenin, 7,4′-*O*-disen, (*S*)-7,4′-*O*-disen, or 7-*O*-sen at 5 µM; control cultures were treated with equivalent volumes of the vehicle of flavonoid dilution (DMSO 0.005%); objective 10x, scale bars = 50 nm. (**B**) Graph showing the relative fluorescence measurement; values are mean ± SEM (n = 3); Data were analyzed using One-way ANOVA followed by Tukey’s multiple comparisons post-test. (*) indicates a statistical difference relative to the negative control (DMSO 0.005%); (#) indicates a statistical difference (*p* < 0.05) between the negative and positive control BSO (1 mM).

Once we determined that flavonoids present antioxidant properties in a cell-free system and are not toxic to astrocytes, we performed experiments to determine the antioxidant mechanisms of naringenin, (*S*)-naringenin, and senecioic acid ester derivatives at low concentrations (5 µM) in astrocytes by determining the capacity to induce GSH, a potent antioxidant [22]. The GHS level was assessed using the monochlorobimane (MCB) test (Figure 4). The results demonstrated a reduction in fluorescence after treatment with BSO, an inhibitor of γ-glutamylcysteine synthetase, used as a positive control. Control cultures (DMSO 0.05%) maintained the basal levels of intracellular GSH when compared with BSO, where the absence of fluorescence indicates glutathione depletion. All flavonoids tested at a concentration of 5 µM increased the levels of GSH after 24 h treatment compared with the control cultures (DMSO 0.005%) (Figure 4).

**Figure 5:**
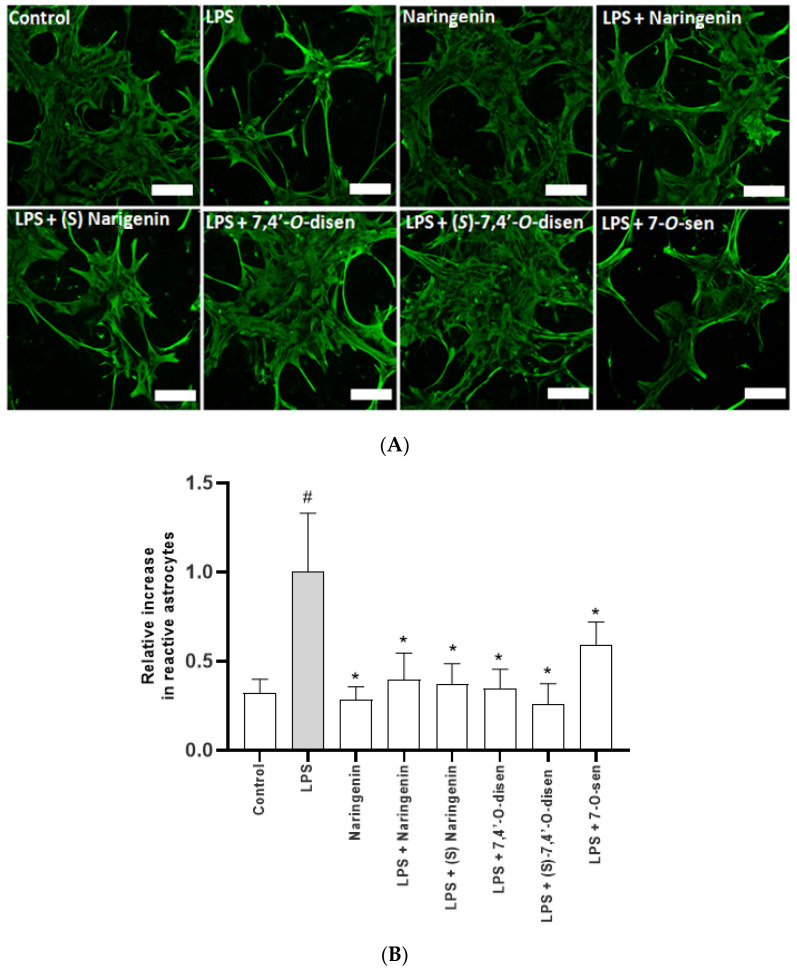
Effect of treatment with the flavonoids naringenin and senecioic acid ester derivatives on astrocyte morphology and reactivity after inflammatory stimulus. Astrocyte cultures were maintained in control conditions (CT, DMSO 0.01%), treated or not with LPS treatment (1 µg/mL) for 24 h, and subsequently treated with naringenin, (*S*)-naringenin, 7,4′-*O*-disen, (*S*)-7,4′-*O*-disen, or 7-*O*-sen (10 µM) after 24 h. GFAP expression, a component of intermediate filaments and a marker of astrocyte reactivity, was analyzed by immunofluorescence. (**A**) Representative photomicrographs of astrocytes expressing GFAP (green) under control conditions (CT, 0.01%) or treated with the flavonoids; obj. 20 × 0.70, scale bar = 100 µm. (**B**) Proportion of reactive astrocytes quantified and plotted; the values are the mean ± SD of fluorescence (n = 5), and the statistical significance was assessed using ANOVA; (*) indicates a statistical difference relative to the positive control LPS (1 µg/mL); (#) indicates a statistical difference (*p* < 0.05) between the negative control (DMSO 0.005%) and LPS-treated cultures.

To investigate the protective potential of flavonoids and senecioic acid ester derivatives in astrocytes and the related structure/activity, we tested the effects of naringenin and its senecioic acid ester derivatives (*S*)-7,4′-*O*-disen or 7-*O*-sen in astrocytes subjected to inflammatory damage with LPS, a component of the Gram-negative bacterial cell membrane known to activate astrocytes and to promote secondary neuronal damage [23]. For this, three parameters were analyzed in the astrocyte cultures subjected to inflammatory damage with LPS (1 µg/mL, 24 h) and treated or not with the compounds: astrocyte phenotype and GFAP expression, NO production, and SOD activity. GFAP is a protein exclusive to astrocytes, is a significant component of the intermediate filaments of the cytoskeleton, and is directly related to cell phenotype associated with astrocyte reactivity after inflammatory stimuli such as with LPS [2,23]. We observed that in the control cultures, the cells presented typical starry to polygonal morphology without showing any signs of cellular damage, with GFAP distributed in the cell body and a short cell process (Figure 5). Significant changes in the morphology of the astrocytes were observed in cultures subjected to inflammatory damage with LPS, characteristic of astrocytic reactivity. The cell retracted the cell body, significantly increasing the cellular process and GFAP expression. On the other hand, in the cultures subjected to LPS damage and treated with the flavonoids naringenin, (*S*)-7,4′-*O*-disen, or 7-*O*-sen, astrocytes assumed phenotypes similar to that observed in the control cultures.

**Figure 6:**
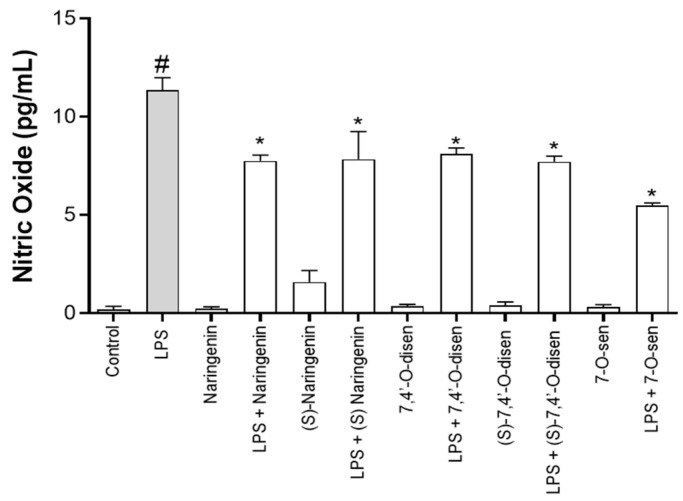
Effects of flavonoids on nitric oxide (NO) production in astrocyte cultures determined by Griess reaction and expressed as nitrite (NaNO_2_) levels. Cultures were exposed or not to LPS (1 µg/mL) for 24 h and treated with the flavonoids naringenin, (*S*)-7,4′-o-diprenylnaringenin, or 7-o-prenylnaringenin at 10 µM or maintained in control conditions (DMSO 0.01%). Results of three independent experiments were analyzed by the Kruskal–Wallis ANOVA test followed by Dunn’s post-test. (*) indicates a statistical difference relative to the positive control LPS (1 µg/mL); (#) indicates a statistical difference (*p* < 0.05) between the negative control (DMSO 0.005%) and LPS-treated cultures.

Considering that astrocytes express inducible oxide nitric synthase (iNOS), an enzyme responsible for nitric oxide (NO) production under inflammatory conditions and associated with neurodegeneration [24,25], we measured NO levels in the medium of the astrocyte cultures under different conditions. The cultures were treated on the first day with LPS (1 µg/mL) for 24 h, and after this period, they were treated with the study molecules (10 µM) for 24 h. LPS treatment induced an increase in NO when compared with the control cultures (DMSO 0.01%). This increase in NO promoted by LPS was significantly reduced by treatments with the flavonoid naringenin and its synthetic derivatives (*S*)-7,4′-*O*-disen or 7-*O*-sen compared with cultures subjected to the inflammatory damage (Figure 6).

**Figure 7:**
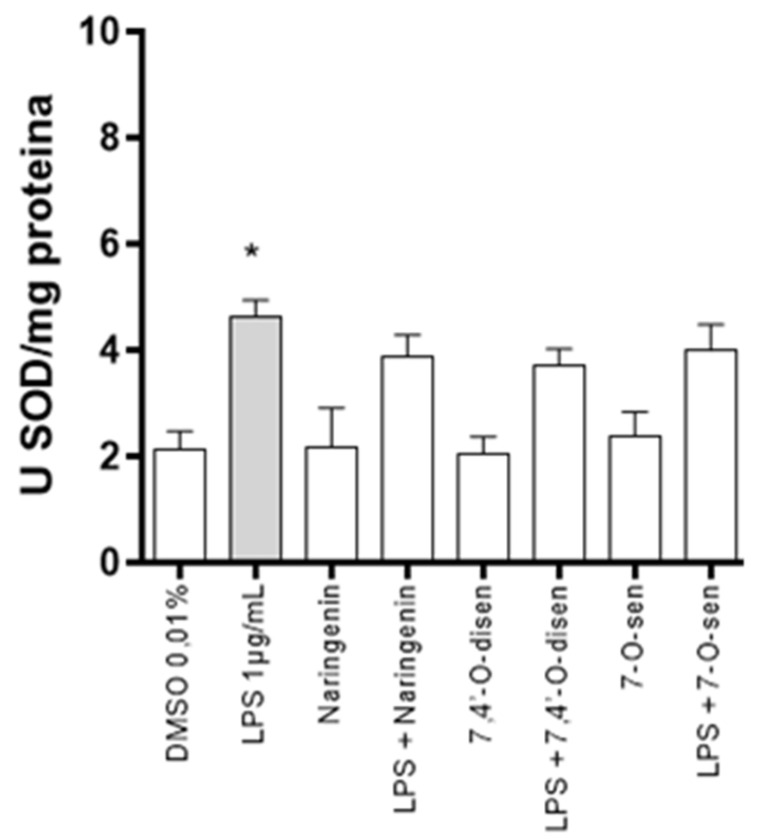
Total of superoxide dismutase one (SOD1) activity in astrocyte primary glia culture. Cultures were exposed or not to LPS (1 µg/mL) for 24 h and treated with the flavonoids naringenin, (*S*)-7,4′-*O*-disen, or 7-*O*-sen at 10 µM or maintained in control conditions (DMSO 0.01%). Results expressed as means and triplicates analyzed by the Kruskal–Wallis ANOVA test followed by Dunn’s post-test. (*) representing *p* < 0.05 with a statistical difference about the control (0.01% DMSO).

Moreover, to investigate the modulation of the astrocytes’ reactivity modulated by naringenin and its synthetic senecioic acid ester derivatives implicated by the modulation of superoxide dismutase (SOD) activity, we performed an assay through cellular homogenates subjected to the nitroblue tetrazolium test (NBT) with subsequent protein and specific SOD1 (EC 1.15.1.1) activity quantification. Astrocytes were treated with LPS (1 µg/mL) for 24 h and then treated or not with flavonoids (10 µM) for an additional 24 h or maintained in control conditions with DMSO (0.01%). LPS treatment induced elevations in SOD1 levels in comparison with control. At the tested conditions, neither naringenin nor its derivatives, (*S*)-7,4′-*O*-disen or 7-*O*-sen, modulated the basal SOD1 activity subsequent to the treatment with LPS (Figure 7).

## 3. Discussion

Naringenin is one of the most abundant flavanones in citrus fruits, and among its diverse properties, it stands out for its antioxidant and anti-inflammatory capacity [8,9,10]. The difference in antioxidant activities between flavonoids is possibly due to the glycosidic portion in position 7 of the A ring of naringin, which results in steric hindrance of the elimination group [26,27,28]. The natural occurrence of senecioic acid esters of flavonoids, which combine the flavonoid moiety and the side chain of the lipophilic prenyl group, is relatively low, which limits the application of these bioactive compounds in pharmaceutical products [29,30]. Therefore, synthesizing these molecules is an excellent way to obtain them. One method of evaluating antioxidant activity is the DHHP assay. However, a disadvantage of the DPPH assay is steric inaccessibility; structurally unfavorable molecules are less likely to access the free radical, resulting in lower DPPH clearance values [19]. The senecioic acid ester derivatives showed a DPPH elimination capacity similar to that of naringenin, notably the senecioic acid esters molecule (*S*)-7,4′-*O*-disen, similar to the standard Trolox. The prenylation of flavonoids enhances their lipophilic capacity. Thus, the more senecioic acid esters in the molecules, the better their interactions with proteins and receptors in biological membranes [6], and the results obtained in this work confirm these mechanisms for the molecule (*S*)-7,4′-*O*-disen with greater lipophilic modification.

Reactive species are essential for the physiological functioning of the brain, and astrocytes actively participate in redox regulation by producing antioxidants and releasing them into the microenvironment [4,5]. Redox changes combined with dysregulation in antioxidant mechanisms lead to oxidative stress and neuroinflammation [31], correlated with reactive astrogliosis [32]; hence, the modulation of astrocyte response has long been considered a therapeutic target for CNS disorders [2,33]. This work demonstrated no significant effects in the mitochondrial function of astrocytes when naringenin, its isomer (*S*)-naringenin, and the senecioic acid ester derivatives 7,4′-*O*-disen, (*S*)-7,4′-*O*-disen, and 7-*O*-sen were adopted at concentrations from 1 to 50 µM, and only a small but significant effect on astrocyte mitochondrial function was evidenced in cultures treated with naringenin or (*S*)-naringenin at the higher concentration (100 µM). GSH is a tripeptide that acts as the central antioxidant molecule in the CNS. Astrocytes can synthesize and release GSH, contributing to redox balance in neurons. Some flavonoids can modulate GSH levels through the influx of cysteine or the modulation of γ-GCS; this limits GSH synthesis, since glutamate and glycine are in relatively high intracellular concentrations [34,35]. Astrocyte-derived GSH plays a strategic role in endothelial cells, promoting stability in the blood–brain membrane’s permeability in a stroke or ischemia model and suppressing the phosphorylation and delocalization of the tight junction [36]. In this work, studying the effects of flavonoids and senecioic acid ester derivatives on GSH levels, it was possible to demonstrate a significant increase in the level of this antioxidant when astrocytes were treated with naringenin or with its senecioic acid ester derivatives. Because astrocytes produce their GSH and provide precursors for its synthesis to neighboring neurons, GSH in the astrocyte is implicated in controlling oxidative stress [36,37], which increases GSH through a feedback mechanism, wherein the antioxidant response is increased because damage is perpetuated. Naringenin and its derivatives modulate GSH levels probably because they reduce the oxidative stimulation/inflammatory cascade [38,39,40].

The lipopolysaccharide (LPS) of *E. coli* is an endotoxin present in the bacterial cell wall that is highly recognized by astrocytes and microglia/macrophages through Toll-like receptor 4 (TLR4), which activates transcription factors in the production of cytokines such as TNF, IL-1, IL-6, and IL-8 in addition to cellular damage due to the generation of ROS and the radical NO; it is therefore well described as a model of neuroinflammation [20,21,23]. Studies have demonstrated that after stimulation with LPS, glial cells increased the production of reactive species. Glial cells, particularly astrocytes, express iNOS, an enzyme responsible for NO production under inflammatory conditions; hence, reactive astrogliosis may contribute to the high NO levels correlated with oxidative stress [25,41,42]. Astrocytes exhibit high expressions of the mitochondrial cofactor-containing enzyme molybdenum sulfite oxidase, which can catalyze nitrite reduction. In the present work, we demonstrated that treatment with LPS induced an increase in NO production in astrocyte cultures; however, its levels were significantly reduced in cultures stimulated by LPS and treated with naringenin and senecioic acid ester derivatives. These findings corroborate other studies that demonstrated the licoflavanone in the murine macrophages RAW 264.7 cell line [43]. Also, the hydroxylated biflavonoid agathisflavone and its monomer apigenin presented a potent neuroprotective effect in neuron–glial cell co-cultures, promoting a reduction in NO production after insults in different cell systems associated with peroxynitrite neutralization responses through electron donation [20,21]. In this study we also observed that LPS treatment induced increased SOD1 activity; however, naringenin and its synthetic senecioic acid ester derivatives did not modulate the SOD1 activity induced by LPS nor the enzyme basal levels. This may indicate that the antioxidant potential of these molecules, naringenin and the senecioic acid ester derivatives, is not via SOD1 but mainly via the modulation of GSH and NO levels [44]. Astrocytes have three SOD isoforms that are in different locations in the cell; their activation after some treatments may be different. Studies demonstrated no changes in SOD2 levels after LPS treatment of astrocytic cells; however, levels of SOD 3, an extracellular isoform, were more highly expressed in astrocytes than in neurons and microglia, and LPS stimulation increased SOD activity in the medium, analyzed by RT-PCR [45]. Further studies could be conducted to clarify the effects of the tested flavonoids on SOD activity.

To better characterize the astrocyte response, we performed immunofluorescence for the intermediate filament protein GFAP. Accumulation of GFAP is a classical marker of reactive astrogliosis during different insults to the CNS, including proinflammatory [46,47] as well as astrocytic hypertrophy and proliferation. We found that inflammatory stimulus by LPS induced significant changes in the morphology of astrocytes, such as hypertrophy and thickening of the cellular process with increased expression of GFAP; the cells presented a more robust and elongated cell process with a very contracted cell body, characteristic of the astrocytic activity expected for LPS. However, in cultures treated with LPS and naringenin or its mono-senecioic acid ester derivative (7-*O*-prenylnaringenin), the astrocytes acquired morphologies similar to and typical of control cultures, with more polygonal phenotypes with GFAP distributed in the cell body and shorter cellular process. Both synthetic 7-*O*-sen and (*S*)-7,4′-disen appeared better at reversing the damage caused by LPS treatment, and this could be attributed to their higher lipophilicity. The cells became less elongated and once again had a small space between them, signs of low cellular damage. Considering that the flavonoids naringenin and its senecioic acid ester derivatives studied here upregulated GSH levels, an antioxidant enzyme that neutralizes oxidative stress, and reduced the NO production induced by inflammatory damage in astrocytes, they could have neuroprotective effects, which will be considered in further studies.

Our results contribute to the understanding of naringenin’s antioxidant effects and mechanism in astrocytes, providing evidence of the most effective effect of senecioic acid ester derivatives on the control of astrocyte reactivity. It is in accord with other studies that demonstrated a more effective effect of 8-prenylnaringenin naringenin from 150 µM in reducing the proliferation of glioblastoma cells, a tumor of astrocytic origin [48]. This effectiveness could be attributed to the prenyl group ensuring better solubility for flavonoid molecules and, therefore, greater permeability through lipophilic cell membranes [49].

## 4. Materials and Methods

### 4.1. Procedures for Flavonoids Purification, Synthesis of Derivatives, and Treatments

All the reagents were obtained from Sigma-Aldrich (St. Louis, MO, USA). All the solvents used in this research study were of analytical grade and used as received from suppliers. Dichloromethane was refluxed with CaH_2_ and distilled prior to use. (*S*)-naringenin was obtained by extraction of naringin from the peel of *Citrus paradisi* (grapefruit) [18] followed by hydrolysis with sulfuric acid in EtOH. The senecioic acid ester derivatives were synthesized by esterification reactions of the respective flavanones with senecioic acid in dichloromethane mediated by *N*,*N*-diisopropylcarbodiimide (DIC) catalyzed by *N*,*N*-dimethyl aminopyridine (DMAP). Unless otherwise stated, all reactions were conducted under a nitrogen atmosphere in flame-dried glassware. Solvents were used as received from suppliers. Melting points were determined on a Microquímica MQAPF 301 hot plate apparatus (São Carlos, Brazil) and are uncorrected. The specific rotations were recorded on a Perkin Elmer 341 LC polarimeter. Infrared (IR) spectra were measured using a Shimadzu IR-Affinity 1 spectrophotometer (Kyoto, Japan). NMR spectra were recorded at 25 °C on a Bruker Avance DRX-400 NMR or DRX-300 NMR spectrometer (Billerica, MA, USA) at 400/300 MHz (^1^H) and 100/75 MHz (^13^C). ^1^H NMR spectra are reported in ppm on the δ scale and referenced to the internal tetramethylsilane. High-resolution mass spectra were acquired by direct infusion of the samples using a Q-Exactive mass spectrometer (ThermoScientific, Bremen, Germany), with H-ESI source used in positive ionization mode, by Federal University of Goiás, Brazil. Column chromatography was performed on silica gel 60 (0.063−0.200 mm, (Al Sigma Aldrich, Darmstadt, Germany). Thin-layer chromatography (TLC) was performed on silica gel 60 F254 (Merck, Darmstadt, Germany) and visualized under a UV lamp (254 nm) or with potassium permanganate stain.

7-*O*-Senecioic ester naringenin (7-*O*-sen): senecioic acid (0.5 mmol, 50 mg) was dissolved in dry CH_2_Cl_2_ (5 mL). Oxalyl chloride (64 µL, 0.75 mmol) was added dropwise. The system was stirred at room temperature, and after gas evolution, it was stirred for another 1 h. The solvent was removed under reduced pressure, and acyl chloride was obtained as a yellow liquid (60 mg, quant.). To a solution of (+/−)-naringenin (136 mg, 0.5 mmol) and Et_3_N (0.07 mL, 0.5 mmol) in dry CH_2_Cl_2_ (4 mL) at −15 °C, a solution of freshly prepared acyl chloride in CH_2_Cl_2_ (0.5 M) was added dropwise (1 mL) for 30 min. The reaction was stirred for another 3 h at the same temperature. Afterward, distilled water (5 mL) was added and stirred for 15 min. The mixture was then diluted in EtOAc (5 mL), and the organic phase was separated. The aqueous phase was extracted with EtOAc (2 × 5 mL), the organic extracts were combined, washed with brine (2 × 5 mL), dried with anhydrous MgSO_4_, filtered, and the solvent evaporated under reduced pressure. The mixture was purified on a chromatographic column (silica gel, EtOAc/Hex, 20%), and 7-*O*-sen was obtained as a white solid (57 mg, 32%). MP: 164.4–165.8 °C; IR (KBr disk) 3437, 3024, 2978, 1717, 1643, 1628, 1585, 1520, 1438, 1123, 1061, 829 cm^−1^; ^1^H NMR (300 MHz, DMSO-d6) δ 11.96 (s, 1 H, OH-5), 9.63 (s, 1 H, OH-4′), 7.34 (d, *J* = 8.5 Hz, 2 H, H-2′; H-6′), 6.81 (d, *J* = 8.5 Hz, 2 H, H-3′; H-5′), 6.32 (s, 2 H, H-6; H-8), 5.91 (s, 1 H, C=CH), 5.57 (dd, *J* = 13.0, 2.6 Hz, 1 H, H-2), 3.48–3.42 (m, 1 H, H-3ax), 2.80 (dd, *J* = 17.2, 2.9 Hz, 1 H, H-3eq), 2.15 (d, *J* = 0.9 Hz, 3 H, CH_3_), 1.97 (s, 3 H, CH_3_); ^13^C NMR (75 MHz, DMSO-d6) δ 198.2 (C-4), 163.0 (C=O), 162.3 (C-5), 162.2 (C-9), 161.8 (C=CH), 158.0 (C-7), 157.9 (C-4′), 128.5 (C-1′; C-2′; C-6′), 115.2 (C-3′; C-5′), 114.2 (C=CH), 105.7 (C-10), 102.7 (C-6), 101.8 (C-8), 78.8 (C-2), 42.3 (C-3), 27.1 (CH_3_), 20.3 (CH_3_); HRMS (ESI/MeOH): *m*/*z* 355.1170 [M + H]^+^ (calculated for C_20_H_19_O_6_, 355.1176).

7,4′-*O*-Disenecioc ester naringenin (7,4′-*O*-disen): naringenin (136.0 mg, 0.5 mmol), DMAP (12.2 mg, 0.1 mmol), and senecioic acid (120.0 mg, 1.2 mmol) were dissolved in dry CH_2_Cl_2_ (2 mL). The system was placed in an ice bath, and then DIC (0.2 mL, 1.4 mmol) was added. It was stirred, and after 30 min, a white precipitate formed. After 25 h of reaction, the mixture was filtered and the solvent evaporated under reduced pressure. The crude was purified with column chromatography (silica gel, 20% EtOAc/Hex) to give 7,4′-*O*-disen as a pale yellow solid (205 mg, 94%). MP: 118.6–118.9 °C; IR (KBr disc) 3060, 2914, 1742, 1638, 1512, 1215, 1192, 1128, 839 cm^−1^; ^1^H NMR (400 MHz, CDCl_3_) δ 11.84 (s, 1 H, OH-5), 7.47 (d, *J* = 8.5 Hz, 2 H, H-2′; H-6′), 7.18 (d, *J* = 8.5 Hz, 2 H, H-3′; H-5′), 6.34 (s, 2 H, H-6; H-8), 5.94–5.93 (m, 1 H, C=CH), 5.88–5.87 (m, 1 H, C=CH), 5.46 (dd, *J* = 13.2, 2.9 Hz, 1 H, H-2), 3.11 (dd, *J* = 17.1, 13.2 Hz, 1 H, H-3ax), 2.88 (dd, *J* = 17.1, 2.9 Hz, 1 H, H-3eq), 2.25 (d, *J* = 1.0 Hz, 3 H, CH_3_), 2.23 (d, *J* = 1.0 Hz, 3 H, CH_3_), 2.01 (d, *J* = 1.5 Hz, 3 H, CH_3_), 2.00 (d, *J* = 1.5 Hz, 3 H, CH_3_); ^13^C NMR (100 MHz, CDCl_3_) δ 196.8 (C-4), 164.6 (C=O), 163.4 (C=O),163.3 (C-5), 162.1 (C-9), 161.5 (C=CH), 160.5 (C=CH), 158.8 (C-7), 151.0 (C-4′), 135.3 (C-1′), 127.2 (C-2′; C-6′), 122.3 (C-3′; C-5′), 114.9 (C=CH), 114.6 (C=CH), 106.0 (C-10), 103.5 (C-6), 101.9 (C-8), 78.7 (C-2), 43.6 (C-3), 27.7 (CH_3_), 27.7 (CH_3_), 20.6 (CH_3_), 20.5 (CH_3_); HRMS (ESI/MeOH): *m*/*z* 459.1410 [M + Na]^+^ (calculated for C_25_H_24_O_7_Na, 459.1414).

(*S*)-7,4′-*O*-Disenecioic ester naringenin ((*S*)-7,4′-*O*-disen): the same procedure above was employed using (*S*)-naringenin instead of (+/−)-naringenin (88%). [α]_D24_-7.0 (c 1.0, CHCl_3_); MP: 125.4–126.0 °C; IR (KBr disc) 3449, 1742, 1637, 1512, 1439, 1215, 1192, 1128, 839 cm^−1^; ^1^H NMR (400 MHz, CDCl_3_) δ 11.84 (s, 1 H, OH-5), 7.47 (d, *J* = 8.5 Hz, 2 H, H-2′; H-6′), 7.18 (d, *J* = 8.5 Hz, 2 H, H-3′; H-5′), 6.34 (s, 2 H, H-6; H-8), 5.93 (s, 1 H, C=CH), 5.87 (s, 1 H, C=CH), 5.47 (dd, *J* = 13.3, 2.9 Hz, 1 H, H-2), 3.12 (dd, *J* = 17.2, 13.3 Hz, 1 H, H-3ax), 2.88 (dd, *J* = 17.2, 2.9 Hz, 1 H, H-3eq), 2.24 (s, 3 H, CH_3_), 2.23 (d, *J* = 0.9 Hz, 3 H, CH_3_), 2.01 (s, 3 H, CH_3_), 2.00 (s, 3 H, CH_3_); ^13^C NMR (100 MHz, CDCl_3_) δ 196.8 (C-4), 164.6 (C=O), 163.4 (C=O),163.3 (C-5), 162.1 (C-9), 161.5 (C=CH), 160.5 (C=CH), 158.8 (C-7), 151.0 (C-4′), 135.3 (C-1′), 127.2 (C-2′; C-6′), 122.3 (C-3′; C-5′), 114.9 (C=CH), 114.6 (C=CH),106.0 (C-10), 103.5 (C-6), 101.9 (C-8), 78.7 (C-2), 43.6 (C-3), 27.7 (CH_3_), 27.6 (CH_3_), 20.6 (CH_3_), 20.5 (CH_3_); HRMS (ESI/MeOH): *m*/*z* 459.1405 [M + Na]^+^ (calculated for C_25_H_24_O_7_Na, 459.1414).

For use in the tests: naringenin, (*S*)-naringenin, and the three senecioic acid ester derivatives 7-*O*-sen, 7,4′-*O*-disen, and (*S*)-7,4′-*O*-disen were diluted in dimethyl sulfoxide (DMSO) at a concentration of 100 mM, forming stock solutions, and kept at −20 °C. As a control condition, dimethyl sulfoxide (DMSO), the vehicle for diluting the molecules in equivalent volume (maximum 0.1%), was considered. The final dilutions of each molecule were prepared according to previous studies with hydroxylated flavonoids [20,21] and diluted directly in a DMEM culture medium free of fetal bovine serum (FBS) for cell treatment.

### 4.2. Biological Experimental Procedures

#### 4.2.1. Cell-Free Screening of the Antioxidant Activity

Firstly, to test if the compounds presented antioxidant activity, we performed the 1,1-diphenyl-2-picrylhydrazyl (DPPH, CAS: 1898-66-4, Sigma Aldrich, Steinheim, Germany) test, a radical scavenging assay, and evaluated the Trolox equivalent antioxidant capacity, performed according to the method described by Baliyan et al. [19] with some modifications for a 96-well microplate. The stock solution of DPPH• radical was prepared in methanol to present absorbance between 0.6 and 0.7 at 517 nm. Reaction volumes of 200 μL, containing 125 μM of DPPH• radical, were prepared in methanol, and 50 μL of different concentrations of flavonoids (1, 10, and 50 μM) were incubated at 25 ± 2 °C for 15 min and read in the microplate reader (Varioskan TM Flash Multimode Reader, Thermoplate Thermo Fisher Scientific, Waltham, MA, USA) at a wavelength of 517 nm. The same procedure was performed with the well-known antioxidant Trolox (CAS: 53188-07-1,Sigma Aldrich, Germany), which was used as standard at equivalent concentrations (1, 10, and 50 μM) [19]. This test was conducted in the dark and at room temperature. The percentage of DPPH• inhibition was calculated using the following equation [19,22,24,27].

% DPPH inhibition = (Ac − At/Ac) × 100

where Ac is the control absorbance and At the test absorbance (flavonoids or synthesis derivatives/Trolox).

#### 4.2.2. Astrocytes Primary Cultures

For astrocyte cultures, the animals were obtained from the Animal Facility of the Institute of Health Sciences (ICS) of the Federal University of Bahia (UFBA), where they were subjected to procedures according to the protocol already well established in the group [50] and approved by the Ethics Committee on the Use of Animals (CEUA) of ICS/UFBA with Protocol No. 6731220818 (ID 000058). In brief, neonatal (P0-2) Wistar rats were decapitated, and their cerebral hemispheres were exposed and aseptically removed. The meninges and blood vessels were removed from the cortex using tweezers. After this procedure, the cortex was mechanically dissociated with a Pasteur pipette. Then, the cells were filtered through a 75 µm sterile Nitex membrane and plated in 75 cm^2^ polystyrene (Falcon^®^ Plates, London, UK) flasks pre-coated with poly-D-lysine (25 µg/mL, Fisher Scientific, Göteborg, Sweden) in PBS, and the cells were suspended in DMEM medium (Dubecco’s Modified Eagle Medium—Cultilab, Campinas, Brazil) supplemented with 10% fetal bovine serum (FBS, Fisher Scientific, Sweden); 44 mM sodium bicarbonate; 100 IU/mL penicillin G, 100 µg/mL streptomycin; 7 mM glucose (Isofarma, Eusébio, Brazil). Cells were kept in a humid chamber with 5% CO_2_ at 37 °C for ten days until they reached confluence. After this moment, the microglia cells were removed from the cultures by stirring at 230 rpm at 37 °C for 3 h, and a fresh DMEM medium (Gibco, Gaithersburg, MD, USA) with supplements was added to the culture and incubated at 37 °C in a humid and controlled atmosphere containing 5% CO_2_ for another 5 days. After this period, the cells were dissociated using a solution of 0.05% trypsin and 0.02% EDTA (Trypsin/EDTA, Sigma Aldrich, Saint Louis, MO, USA, 9002077) diluted in PBS free of calcium and magnesium for 5 min in an oven and re-plated with the aid of counting in a Neubauer chamber at a density of 7.5 × 10^4^ cells/well in 96-well, 24-well, or 10 mm diameter polystyrene plates for the different assays.

#### 4.2.3. Analysis of Cell Viability by the MTT Test

The effect of flavonoids on astrocyte viability was evaluated through mitochondrial activity after 24 h treatment. The 3-(4,5-dimethylthiazol-2-yl)2,5-diphenyltetrazolium bromide test (MTT test, Thermo Fisher, Waltham, MA, USA) was performed, which is based on the conversion of this yellow tetrazolium salt into formazan crystals of violet color through the mitochondrial dehydrogenase enzymes of viable cells. Astrocytes were plated in 96-well plates, and after 24 h, the medium was replaced with fresh medium containing the flavonoid naringenin, (*S*)-naringenin and its senecioic acid ester derivatives at concentrations of 1 to 100 µM, or the vehicle DMSO at the highest equivalent volume (0.1%). The concentration adopted ranged from that of a previous study with structurally similar flavonoids, which demonstrated neuroprotective or neurotoxic effects [20,21].

After 24 h treatments, 100 µL of MTT solution (1 mg/mL) diluted in DMEM was added to each well of the plates, which were incubated in a humid chamber with 5% CO_2_ at 37 °C for 2 h. After exposure to MTT, 100 µL/well of a lysis buffer solution (20% SDS and 50% DMF, pH 4.7) was added to the plates to dissolve the formazan crystals at room temperature. The absorbance reading was performed after 18 h (overnight) in a microplate reader Varioskan TM Flash Multimode Reader (Thermoplate Thermo Fisher Scientific, Waltham, MA, USA) at a wavelength of 595 nm. Cell viability was represented by the percentage of viable cells related to control cultures treated with DMSO (0.1%), which was considered 100%. No statistical difference was observed between negative control cultures and those exposed to only the vehicle control (DMSO 0.1%). Experiments were performed in octuplicate in three independent experiments.

#### 4.2.4. Analysis of Glutathione (GSH) Depletion

To investigate antioxidant properties of naringenin, (*S*)-naringenin, and its senecioic acid ester derivatives in astrocytes, we evaluated their effects on the levels of glutathione, which is a low-molecular-weight molecule composed of three amino acids (glutathione-γ-L-glutamyl-L-cysteinyl-glycine) and considered the most important antioxidant synthesized in cells, including in astrocytes, serving as substrate for peroxidases and is conjugated with free radicals, being a key regulator of stress-sensing pathways [51,52] that boosts the cellular metabolic system’s defense against insult. Monochlorobimane (MCB) (Sigma Aldrich, St. Louis, MO, USA) was used to assess reduced glutathione (GSH). A stock solution of MCB was prepared in 100 mM in methanol p.a. and stored at −20 °C in the dark. ADL-buthionine-*SR*-sulfoximine (BSO, 1 mM), a specific GSH synthesis inhibitor, was used as a positive control. Astrocytes were plated in 24-well plates (TPP), and after 24 h, the cells were treated with BSO, maintained in control conditions (DMSO 0.005%), or treated with the flavonoids at 5 µM, a nontoxic and intermediary concentration between the effective antioxidant concentrations for all compounds in cell-free systems (DPPH assay). After 24 h treatments, the cultures were washed three times with phosphate-buffered saline solution free of Ca^2+^ and Mg^2+^ for 30 s (PBS: 2.7 mM KCl, 1.5 mM KH_2_PO_4_, 8 mM Na_2_HPO_4_, and 136.9 mM NaCl, pH 7.0) and incubated with 50 µM MCB in 10 mL of culture medium for 40 min. After the incubation, the cells were washed with PBS. Free GSH was detected using a fluorescence microscope (Leica, Wetzlar, Germany, DFC7000), coupled with an AxioCamHRm camera (Leica, Nussloch, Germany). The photographs were taken with an exposure time of 211 ms for all samples, where 8 randomized fields were photographed for analysis in each condition, and the generated images were analyzed using ImageJ 1.33u Software (Wayne Rasband, National Institutes of Health, Kensington, MD, USA).

#### 4.2.5. Analysis of Astrocyte Phenotype and Reactivity

With a view to investigating the protective potential of flavonoids and senecioic acid ester derivatives in astrocytes and the related structure/activity, we tested the effects of naringenin and its senecioic ester derivatives (*S*)-7,4′-*O*-disen or 7-*O*-sen on astrocytes subjected or not to inflammatory damage with the lipopolysaccharide (LPS) from *Escherichia coli*. LPS is a toxin known for its capacity to interact with Toll-like receptors expressed in astrocytes and to activate inflammatory pathways that give rise to the expression of numerous proinflammatory genes, and consequently, can contribute to secondary neuronal damage [20,21,23,53]. For experiments, astrocytes were subjected to inflammatory damage with LPS (1 µg/mL 24 h), and treated or not with the compounds at a concentration of 10 µM, also a nontoxic and effective antioxidant concentration for naringenin and the tested derivatives in cell-free DPPH system.

#### 4.2.6. Analysis of Astrocyte Phenotype by Immunofluorescence

Glial fibrillary acidic protein (GFAP) is a component of intermediate filaments specific to the cytoskeleton of astrocytes and is directly related to cell phenotype associated with astrocytes’ reactivity [1,2,54], and a change in its expression and, consequently, in astrocyte morphology, is considered one of the primary features of astrocytes’ reaction to different insults, including inflammation with LPS. Hence, we investigated the effect of naringenin, (*S*)-7,4′-*O*-disen, and 7-*O*-sen on GFAP expression in astrocytes subjected to inflammatory damage with LPS. For this, astrocytes (7.5 × 10^4^ cells/cm^2^) were plated in 6-well chamber slices, pre-sensitized with poly-ornithine (50 μg/mL, Sigma-Aldrich P0671), and 24 h later, the cells were treated with LPS (1 µg/mL, 24 h), treated for 24 h with the compounds (10 µM), or maintained in control conditions (DMS 001%) and then processed for immunofluorescence analysis. After time treatments, cultures were washed three times with PBS and fixed with ice-cold methanol for 10 min at −4 °C. Afterward, the methanol was discarded, and the plates containing the fixed cells were stored at −8 °C until immunofluorescence was carried out. Cells were rehydrated with PBS for 30 min under agitation in a shaker. Afterward, non-specific bindings were blocked by pre-incubation of coverslips for 30 min with 5% BSA diluted in PBS. Cells were incubated with primary antibodies (rabbit anti-GFAP polyclonal; DAKO Z0334; 1:500, Merck, Darmstadt, Germany) and diluted in 1% BSA in PBS for 3 h, following the dilution instructions according to the manufacturer. After this, the cells were washed 3 times with PBS and incubated with secondary antibodies (goat anti-rabbit IgG-polyclonal conjugated with Alexa Fluor 488, Invitrogen A11008, 1:1000) for 1 h at room temperature and diluted in 1% BSA in PBS, following the concentration indicated by the manufacturer. Three new washes with PBS were performed, and the nuclear chromatin was stained with the fluorescent DNA intercalating agent 4′6-diamidino-2-phenylindole (5 μg/mL; DAPI, Invitrogen—Probes Molecular, Eugene, OR, USA) for 5 min in a dark chamber at room temperature. After these steps, analysis was performed by fluorescence microscopy (Leica DCF7000T, coupled to the AxioCam HRm camera Leica, Nussloch, Germany), where 6 randomized fields were photographed for each treatment and culture, and the generated images were analyzed using ImageJ 1.33u Software (Wayne Rasband, National Institutes of Health, USA).

### 4.3. Nitric Oxide Production

Astrocytes express inducible nitric oxide synthase (iNOS), an enzyme responsible for nitric oxide (NO) production under inflammatory conditions, and reactive astrogliosis, which may contribute to high nitric oxide levels correlated with oxidative stress [55]. Hence, in this study, we investigated the effect of naringenin, (*S*)-7,4′-*O*-disen, and 7-*O*-sen on NO production in astrocytes subjected to inflammatory damage with LPS. The production of NO was evaluated through the accumulation of sodium nitrite (NaNO_2_) in the culture medium using a colorimetric test based on the Griess reagent [55]. For this, astrocytes (7.5 × 10^4^ cells/cm^2^) were plated in 24-well plates, and 24 h later, the cells were treated with LPS (1 µg/mL/24 h) and treated for 24 h with the compounds (10 µM) or maintained in control conditions (DMSO 0.01%). After these time treatments, samples of 50 µL were collected, and equal volumes of culture medium and Griess reagent (1% sulfanilamide, 0.1% *N*-(1-naphthyl) ethylenediamine dihydrochloride, and 2% phosphoric acid—Sigma Aldrich, Saint Louis, MO, USA) were mixed (1:1 ratio). The mixture was incubated for 10 min at room temperature. The absorbance was measured at 540 nm using a microplate reader (Varioskan TM Flash Multimode Reader, Thermoplate). Nitrite concentrations in the samples were determined based on a sodium nitrite standard curve (1.26–100 mmol/L NaNO_2_). The experiments were performed in triplicate in three independent astrocyte cultures.

### 4.4. Superoxide Dismutase (SOD) Activity

Astrocytes actively participate in redox regulation by producing antioxidants, including by increasing the expression and activity of SOD. To determine the SOD’s (EC 1.15.1.1) activity, a standard protocol was used [41]. The cells were plated in 100 mm plates (7.5 × 10^4^ cells/cm^2^). After 24 h, the cultures were treated with flavonoids at 10 µM or maintained in control conditions (DMSO 0.01%). After the time treatment (24 h), the culture media was discarded, and the cells were washed 3 times with 5 mL of PBS for 30 s. Most of the buffer was removed, leaving 1.5 mL. With the aid of a cell scraper, the cells were scraped from the plate’s surface, collected, and centrifuged in a refrigerated centrifuge at 1500 rpm for 10 min. After this step, the supernatant was discarded, the cell pellets were resuspended in 100 µL of PBS (pH 7.8), and the samples were sonicated (Qsonica Q55-110, Newtown, CT, USA) with an amplitude of 40% for 30 s for 2 cycles in an ice bath. The cell samples were stored at –70 °C and were then subjected to enzymatic analysis. Total SOD activity was determined by measuring its ability to inhibit the photochemical reduction of nitroblue tetrazolium chloride (NBT) by O_2_^•−^. The initial reaction rate was determined by absorbance at 560 nm using a microplate reader (Varioskan TM Flash Multimode Reader, Thermoplate Thermo Fisher Scientific, Waltham, MA, USA). Initially, 180 μL of the reaction mixture (50 mM PBS (pH 7.8), 14 mM methionine, NBT 75 mM, 2 mM riboflavin, 0.1 mM EDTA) was mixed with 6 μL of the cell sample. Then, the reaction was started by irradiating a 9W fluorescent lamp at 25 °C for 9 min (time established after carrying out the reagent mix’s consumption curve). The inhibition of the NBT production was proportional to the SOD activity present in the sample. The enzyme activity was expressed as SOD activity units (U) per mg of total protein. A SOD activity unit is expressed as the amount of enzyme needed to cause 50% inhibition of NBT reduction under experimental conditions. Four independent experiments were performed.

### 4.5. Statistical Analysis

Three independent experiments were carried out where the generated data received appropriate statistical treatment using the GraphPad Prism software, version 5.0 or 6.0 for Windows (GraphPad Software, San Diego, CA, USA), expressed as mean ± standard deviation or median ± percentiles. The *p* values adopted as statistically significant in the analyses were those below 0.05, with * *p* < 0.05. The results obtained with normal distribution were represented by means (Shapiro–Wilk normality tests or D’Agostino and Pearson test), chosen as parametric statistical tests used to compare treated groups and controls. Non-parametric tests were used to represent the results represented by the median (non-normal distribution).

## 5. Conclusions

The results obtained in this work demonstrated that the senecioic acid ester derivative (*S*)-7,4′-*O*-disen presented a more significant antioxidant potential, similar to Trolox, and naringenin and other senecioic acid ester derivatives presented moderate antioxidant activity, in the analysis of the free radical scavenging potential from DPPH. It was found that naringenin and the senecioic acid ester derivatives did not induce toxicity or morphological changes in GFAP-positive cells from primary astrocyte culture, presented antioxidant action involving GSH induction, and presented anti-inflammatory effects characterized by a reduction in the inflammatory mediator NO, an effect related to the modulation of structural features of astrocyte reactivity. Among the tested molecules, naringenin and disenecioic ester naringenin presented the most beneficial impact on all parameters evaluated. This confirmed the hypothesis that flavonoids and senecioic acid ester derivatives have antineuroinflammatory and antioxidant properties. The results found here serve as a basis for additional studies in view of the characterization of the potential neuroprotective effects and mechanisms.

## Data Availability

We provide additional data from this work, which can be found as Supplementary Materials, supporting the reported results.

## Supplementary Materials

The following supporting information can be downloaded at: https://www.mdpi.com/article/10.3390/ijms26052215/s1.
