# Supplementary material for: Synthesis of Naringenin and Senecioic Acid Ester Derivatives and Biological Evaluation of the Astrocyte Antioxidant Mechanism and Reactivity After Inflammatory Stimulus
Source: Int J Mol Sci. 2025 Feb 28;26(5):2215. doi: 10.3390/ijms26052215 (PMC11900193; doi:10.3390/ijms26052215)
Supplement: Supplementary file 1 [file ijms-26-02215-s001.zip › ijms-3492561-supplementary.pdf]

Supporting Information

Synthesis of naringenin senecioic acid esters derivatives and biological evaluation on astrocyte antioxidant mechanism and reactivity after inflammatory stimulus

Janaína Ribeiro Pereira Soares [1], Cleonice Creusa dos Santos [1], Lucas Matheus Gonçalves de Oliveira[1], Heráclito Rocha Neto[1], Maurício Moraes Victor [2], Elivana Lima França[3], Maria de Fátima Dias Costa [1,4], Silvia Lima Costa [1,4*] and Juciele Valeria Ribeiro de Oliveira [1,*]

1. Laboratory of Neurochemistry and Cellular Biology, Institute of Health Sciences, Federal University of Bahia, Av. Reitor Miguel Calmon S/N, Salvador 40231-300, Brazil;

2. Department of Organic Chemistry, Institute of Chemistry, Federal University of Bahia, Salvador 40170-115, Brazil;

3. Federal Institute of Bahia, Campus Vitória da Conquista, Vitória da Conquista 45078-300, Brazil;

4. National Institute of Translational Neuroscience (INNT), Brazil.

*Correspondence: costasl@ufba.br (S.L.C.); juciele.valeria@ufba.br (J.V.R.d.O.);

Table of contents

1. Spectra S2-S4

1. Spectra

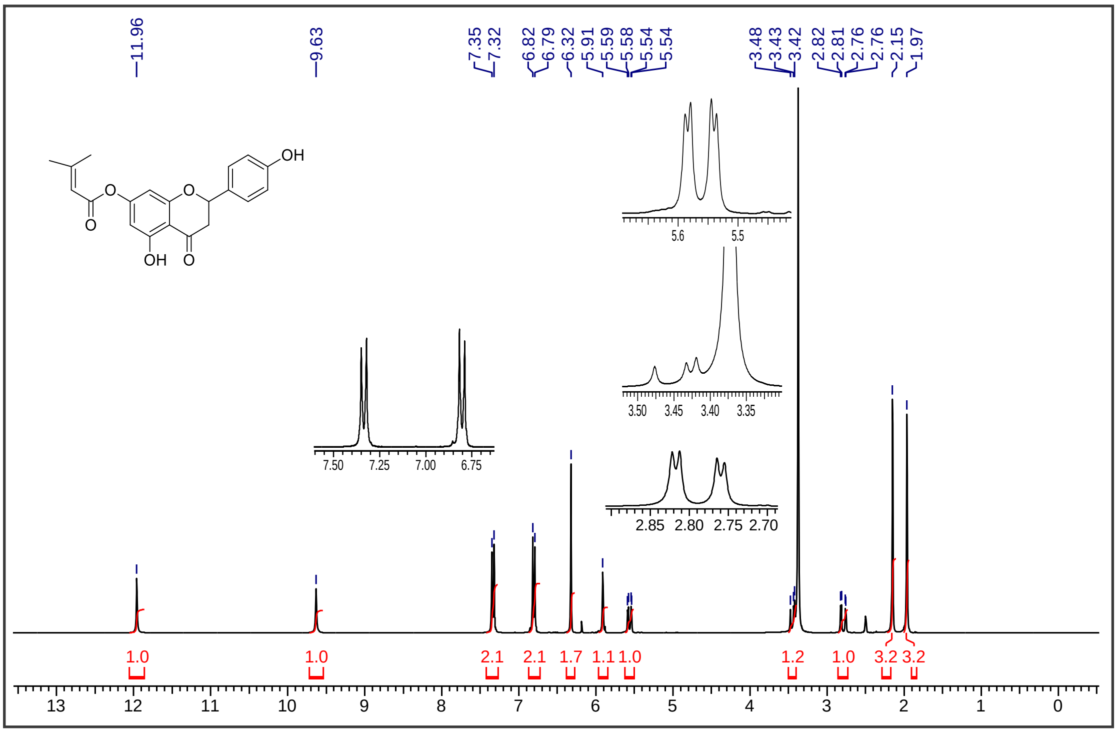

^{1}H NMR of 7-*O*-sen (DMSO-d$_6$, 300 MHz)

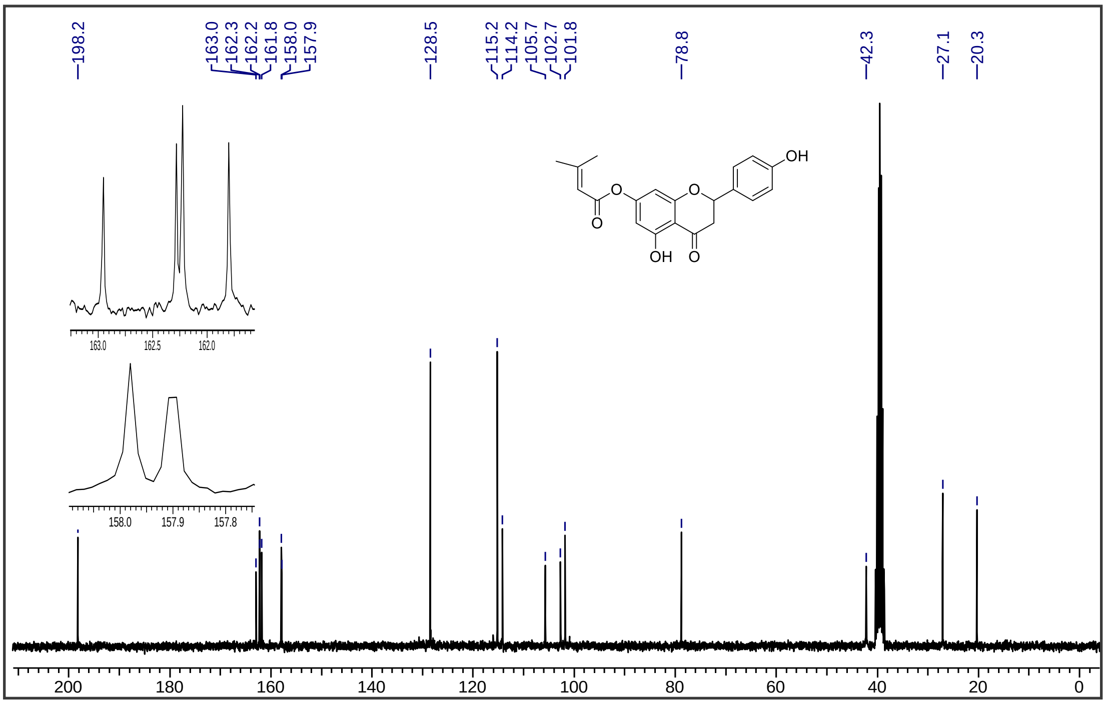

^{13}C NMR of 7-*O*-sen (DMSO-d$_6$, 75 MHz)

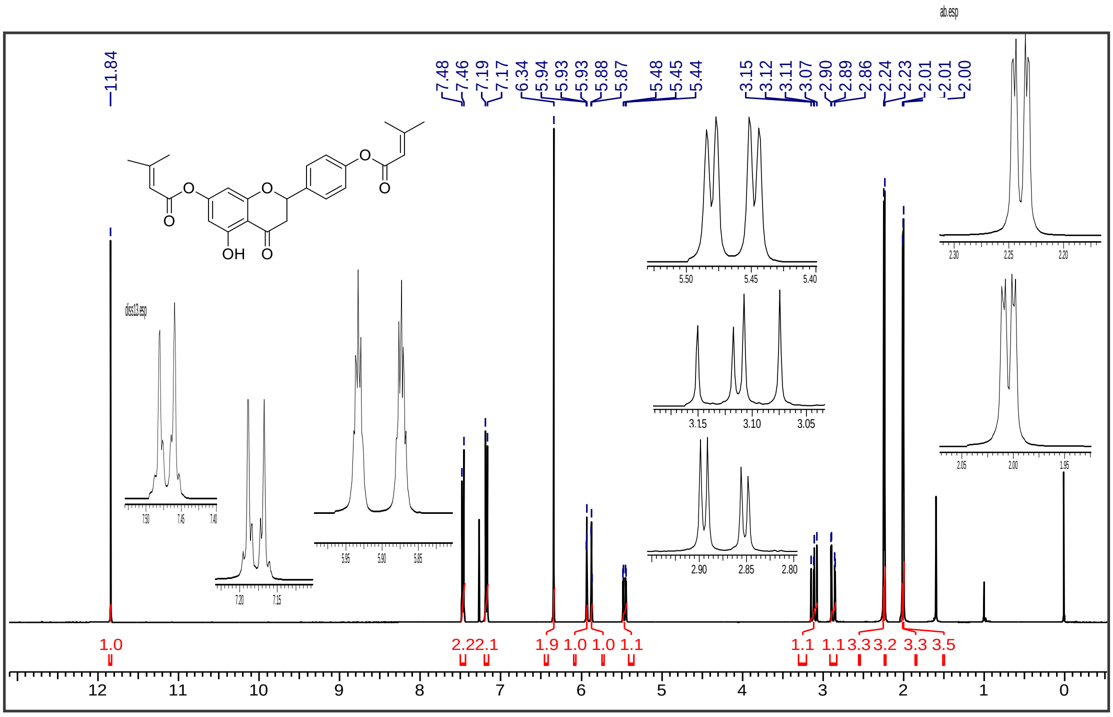

^{1}H NMR of 7,4'-*O*-disen ($CDCl_3$, 400 MHz).

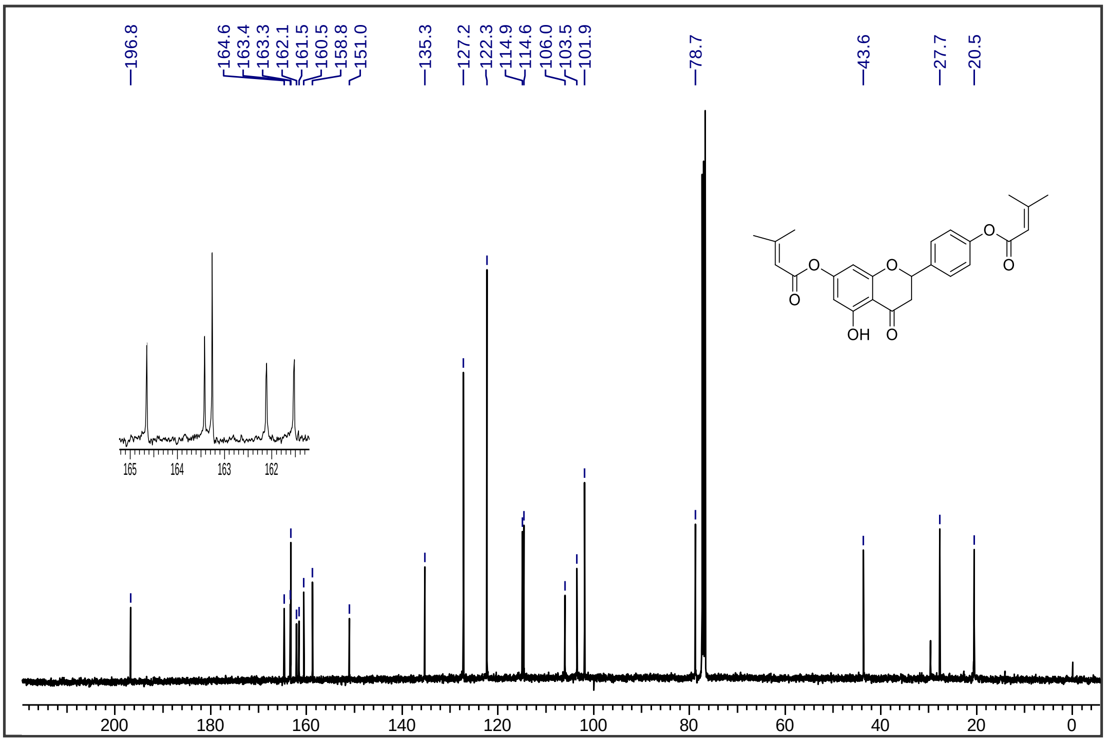

^{13}C NMR of 7,4'-*O*-disen ($CDCl_3$, 100 MHz).

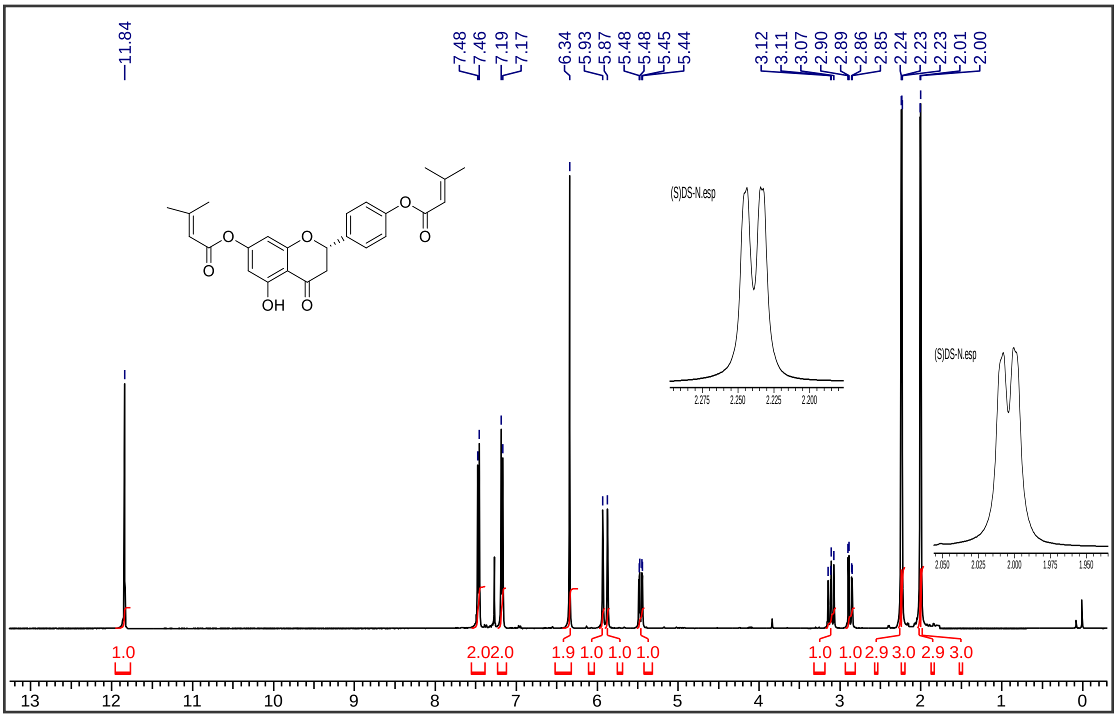

^{1}H NMR of (*S*)-7,4'-*O*-disen (CDCl$_3$, 400 MHz).

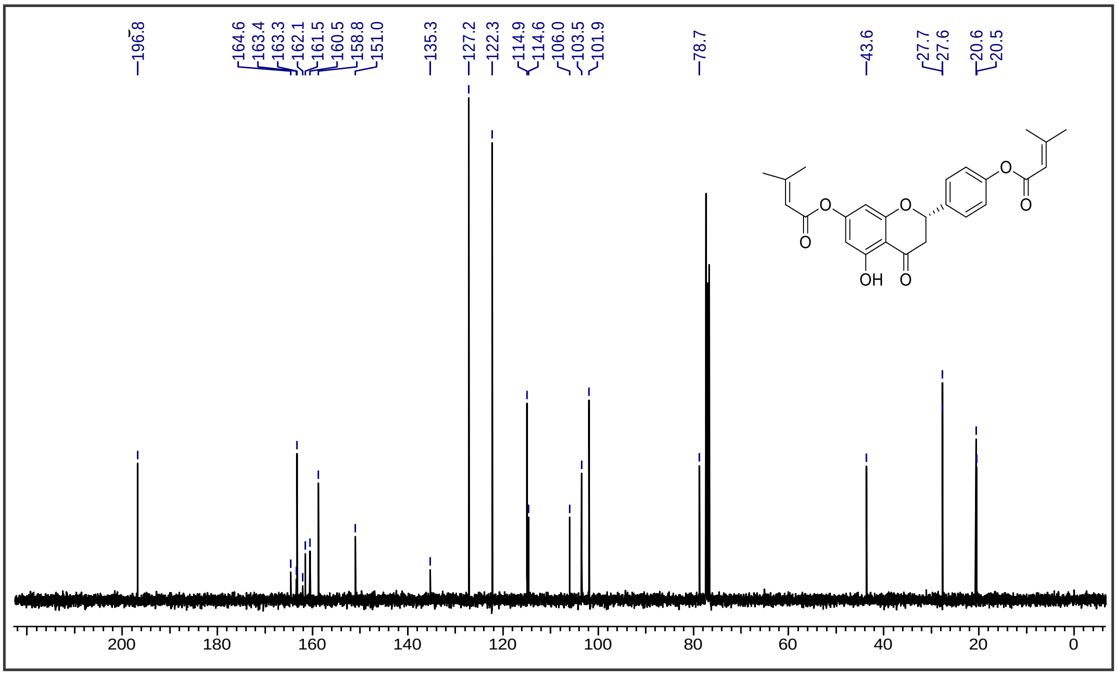

^{13}C NMR of (*S*)-7,4'-*O*-disen (CDCl$_3$, 100 MHz).
